# DYADIC EFFECTS OF PERSONALITY AND SEXUAL BEHAVIOR IN OLDER ADULTHOOD

**DOI:** 10.1093/geroni/igad104.1714

**Published:** 2023-12-21

**Authors:** William Chopik

**Affiliations:** Michigan State University, East Lansing, Michigan, United States

## Abstract

Personality traits are associated with important life, work, and relational outcomes across the lifespan. Although personality has been implicated in models of relationship functioning, there has been relatively little research matching individual and partner personality characteristics to the frequency, quality, and variety of sexual experiences, particularly in older adulthood. The current study examined individual and spousal effects of the Big Five personality traits on sexual outcomes among 953 older opposite-sex couples followed from 2010/2011 to 2015/2016 as part of the National Social Life, Heath, and Aging Project (NSHAP; Mage = 70.60, SD = 7.92; 74.2% White, 12.2% Hispanic, 11.1% Black, and 2.5% other). Individual and spousal conscientiousness was associated with more frequent dyadic and solitary sexual activity and being part of a physically pleasurable and emotionally satisfying relationship. Those with higher levels of neuroticism and those married to spouses high in neuroticism expressed a lower preference for physical/sexual touch and cuddling, were less likely to report having a physically pleasurable and emotionally satisfying relationship, and were more likely to avoid having sex because of physical problems (and report more pain during sex). Longitudinal analyses suggested that most of these associations wavered in strength over time. The findings are discussed in the context of how relationship processes might mediate the link between personality characteristics and sexual outcomes or whether sexual outcomes promote relationship-enhancing characteristics depending on people’s personalities.

